# Maternal, Fetal, and Labour Outcomes of Dupilumab Use for Atopic Dermatitis During Pregnancy: A Systematic Review

**DOI:** 10.1177/12034754241290806

**Published:** 2024-10-20

**Authors:** Chirag R. Chopra, Mahak Sharma, Mahtab S. Gill, Victoria Del Balso, Noor Sakka, Mohannad Abu-Hilal

**Affiliations:** 1 School of Medicine, Queen’s University, Kingston, ON, Canada; 2 Faculty of Science and Horticulture, Kwantlen Polytechnic University, Surrey, BC, Canada; 3 Faculty of Medicine, University of British Columbia, Vancouver, BC, Canada; 4 Faculty of Health Sciences, McMaster University, Hamilton, ON, Canada; 5 School of Medicine, University of Jordan, Amman, Jordan; 6 Division of Dermatology, McMaster University, Hamilton, ON, Canada

**Keywords:** atopic dermatitis, dupilumab, dupixent, pregnancy, biologic

## Abstract

Atopic dermatitis is a chronic complex inflammatory disease that significantly impacts maternal well-being and quality of life during pregnancy, warranting effective therapeutic interventions that prioritize maternal health and fetal safety. Dupilumab is approved for moderate-to-severe atopic dermatitis, but limited data exist regarding its safety during pregnancy. We conducted a systematic review to review and analyze maternal, fetal, and labour outcomes in patients receiving dupilumab for atopic dermatitis during pregnancy. Comprehensive searches were conducted using databases including OVID, Scopus, and Web of Science, covering studies published until May 2024. Our search yielded 285 studies, of which 13 met the eligibility criteria. These studies included 68 patients with 69 pregnancies, revealing 58 live births and 11 spontaneous abortions. Dupilumab therapy was administered continuously throughout pregnancy in 22.2% of cases, while 77.8% received intermittent treatment. Maternal atopic dermatitis outcomes showed significant improvement in disease severity. Most pregnancies (86.3%) progressed without complications. Labour-associated outcomes varied, with 82.4% of women undergoing vaginal deliveries. The majority of births occurred at full term (82.5%), with a mean gestational age of 38.4 weeks. Fetal outcomes demonstrated a normal birth weight in 92.3% of cases, with no reported congenital defects. Our review suggests that dupilumab use during pregnancy is associated with improvement of atopic dermatitis and low or minimal risk of major adverse outcomes in treated patients or their newborns. Prospective studies with long-term follow-up are warranted to confirm the safety of dupilumab in this population.

## Introduction

Atopic dermatitis (AD) is a chronic inflammatory skin disease with a prevalence of 10% in the adult population.^1,2^ Greater than 50% of women with AD are expected to experience a deterioration of symptoms during pregnancy.^3-5^ Complications of moderate to severe AD can pose risks to pregnant women and fetal health, including bacterial (eg, *Staphylococcus aureus*) or herpetic infections (eg, eczema herpeticum).^6-8^ Moreover, in 1 Danish cohort of 10,688 births from women with AD, an increased risk of premature rupture of membranes and staphylococcal neonatal septicemia were observed.^8^

Treating AD during pregnancy typically involves the use of topical corticosteroids and narrowband ultraviolet-B therapy given their well-documented safety profiles. In severe cases, cyclosporine may be required. However, some individuals may not tolerate or respond well to these therapies, necessitating the consideration of other effective and safe options.^9^

Dupilumab, a human monoclonal antibody of the immunoglobulin G (IgG) 4 subclass, targets the IL-4 alpha receptor, which plays a crucial role in the IL-4 and IL-13 signalling pathways. It is approved for treating moderate-to-severe AD in patients aged 6 months and older. Unlike cyclosporine and other traditional systemic therapies, dupilumab acts as an immunomodulator rather than an immunosuppressant, making it a safer option for managing AD during pregnancy.^10-13^ There are, however, limited data on the safety of dupilumab use during pregnancy and its effect on maternal-fetal health. Similar to other IgG antibodies, dupilumab is anticipated to cross the placental barrier, particularly during the 2nd and 3rd trimesters, posing potential exposure to the developing fetus.^14,15^

The objective of this review is to analyze published data on the maternal, fetal, and labour outcomes for patients receiving dupilumab during pregnancy for AD. This review is crucial for filling gaps in current knowledge and providing better-informed decisions for patients with moderate to severe AD who are pregnant or considering pregnancy.

## Methods

This study was conducted in accordance with the Preferred Reporting Items for Systematic Reviews and Meta-Analyses (PRISMA) guidelines and was registered with the International Prospective Register of Systematic Reviews (PROSPERO—CRD42024532187). Comprehensive searches were conducted using databases including OVID (MedLine, Embase, AMED, PsychINFO, JBI EBP Database), Scopus, and Web of Science, covering studies published until May 2024. The search string used was as follows: ((“dupilumab” or “dupixent”) and (“pregnancy” or “pregnant” or “gestation” or “antenatal” or “maternity” or “pregnant patient” or “pregnant woman” or “pregnant lady” or “gravida”) and (“atopic dermatitis” or “atopic eczema” or “AD”).

Case reports, case series, randomized controlled trials, prospective studies, pharmacovigilance and drug assessment reports, and cross-sectional studies were included. Exclusion criteria comprised review articles, book chapters, opinion pieces, conference/poster abstracts, research focusing on conditions other than AD, and theses. Pregnant patients treated with dupilumab solely during the preconception or postpartum periods, and pregnant patients treated with dupilumab for non-dermatologic indications or non-AD dermatological diseases were also excluded. Inclusion criteria during the title and abstract review included patients who were treated with or were exposed to dupilumab during pregnancy for AD. Two researchers screened articles evaluating inclusion, exclusion, and source quality criteria. Source quality was assessed using the modified Oxford Centre for Evidence-based Medicine scale. Disagreements were resolved by a third researcher for consensus.

## Results

The search yielded 285 studies. After removing 166 duplicate articles, 119 were screened, and 13 studies met the eligibility and were included. The 13 reports were as follows: 8 case reports, 1 case series, 3 retrospective studies, and 1 drug assessment report including cases from a pharmacovigilance report by a European Medicines Agency (EMA).^16^ A summary of the review process is described in the PRISMA Flow Chart (Supplemental Figure 1).

Our study identified 68 patients undergoing 69 pregnancies, including 1 patient followed over a span of 2 years with 2 consecutive pregnancies. A total of 69 pregnancies with 58 live births (84.1%) included 1 pregnancy resulting in twins, and 11 spontaneous abortions (15.9%). All patients with reported disease onset were diagnosed with AD prior to pregnancy (Supplemental Table 2). Of all reported patients, dupilumab therapy was given continuously throughout the pregnancy in 22.2% of patients, while in 77.8%, it was administered intermittently or during part of the pregnancy. The mean maternal age was 33.7 years (range: 28-41). Among the reports with detailed continuous treatment plans, all patients adhered to the approved dose of dupilumab of 300 mg every 2 weeks, except for 1 instance where the dosage was adjusted to 300 mg every 3 weeks due to ocular side effects (eye irritation) which resolved with continued treatment.^17,18^ A total of 49 patients reported undergoing dupilumab treatment before pregnancy and 4 reported starting during their pregnancies, of which 1 patient began the first treatment during their 1st trimester, 2 during their 2nd, and 1 during their 3rd (Supplemental Table 3). When reported, all patients received the approved dose of dupilumab.

### Maternal Outcomes

All patients experienced significant alleviation of AD severity reflected by improvement in Eczema Area and Severity Index, Investigator Global Assessment, Dermatology Life Quality Index scores, and/or through self-reporting measures. One patient, who presented with diagnoses of AD, hyper IgE syndrome, and ulcerative colitis, experienced complete resolution of skin lesions over the span of several months, accompanied by significant improvements in her SCORing Atopic Dermatitis and Peak-Pruritus Numerical Rating Scale scores. Moreover, there was notable relief in the patient’s respiratory symptoms without exacerbation of her gastrointestinal symptoms.^19^ Of the pregnancies analyzed, 86.3% progressed without complications. Seven pregnancies exhibited one or more complications such as ocular surface disease (n = 3, 4.4%), gestational diabetes (n = 3, 4.4%), mild arthralgia (n = 1, 1.5%), post-partum hemorrhage (n = 1, 1.5%), and 1 case of oligohydramnios (n = 1, 1.5%).

### Labour-Associated Outcomes

Seventeen out of the total 69 pregnancies reviewed explicitly reported the type of birth (vaginal or cesarean). Of these, 14 patients (82.4%) had vaginal births, while 3 patients (17.6%) had cesarean births. Overall, 7 pregnancies experienced labour-related complications including cesarean sections and assisted deliveries but excluding spontaneous abortions. Most births occurred at full term (82.5%), pre-term (17.5%), and no post-term births were recorded. The mean gestational age at birth was 38.4 weeks with a range from 35 to 41 weeks.

### Fetal Outcomes

A total of 58 live births included 1 pregnancy resulting in twins and 11 cases of spontaneous abortions. Most spontaneous abortions were cited in the European Medicines Agency’s 2017 report on dupilumab.^16^ The average reported birth weight across the cases was 2869.6 g. Normal birth weight accounted for 94% of babies (2500-4500 g), 6% had low birth weight (less than 2500 g), and no reports of high birth weight (greater than 4500 g) were recorded. A notable case of intrauterine growth restriction coupled with a breech position was documented at 38 weeks of gestation, necessitating a cesarean procedure.^20^ However, a healthy newborn was delivered despite these challenges. Another case involved a fetus initially categorized as small for gestational age during pregnancy; however, the baby was ultimately delivered at full term with a healthy birth weight.^21^ Overall, observational reports claim an excellent range of fetal outcomes in women exposed to dupilumab treatment during pregnancy.

## Discussion

AD is a prevalent condition during pregnancy and uncontrolled disease can pose risks to maternal and fetal health, potentially leading to serious complications such as eczema herpeticum and severe cutaneous *S. aureus* infections.^6-8^ Untreated AD may also result in anxiety, depression, and significant impairment of quality of life.^22-26^

Treatment of AD during pregnancy typically includes topical corticosteroid agents and narrowband ultraviolet-B therapy, and in severe cases, cyclosporine. However, AD could be refractory to such therapies requiring more effective agents.^27^ Overall, all patients receiving dupilumab in our review achieved significant improvement in AD symptoms. Dupilumab is a human monoclonal IgG4 antibody and is expected to pass through the placental barrier with the highest likelihood in the 2nd and 3rd trimesters.^28,29^ Dupilumab is classified as a pregnancy category “B1” in the Australian Therapeutic Goods Administration’s classification system for pregnancy.^30,31^ This classification indicates no increase in the occurrence of birth defects or adverse effects on fetal development based on use in a limited number of pregnant individuals. The data are supported by studies in cynomolgus monkeys where no adverse effects related to pregnancy were observed.^16,32^ In addition, studies in mice did not find any impact on fertility or developmental outcomes.^32^

Among the 69 pregnancies analyzed in this study; most patients experienced no complications during pregnancy while on dupilumab treatment (86.3%). However, 11 cases of spontaneous abortions were reported. Six of these cases were included in the EMA pharmacovigilance database.^16^ Two of these cases were individuals who exhibited one or more known major risk factors for abortion, such as elevated parathyroid hormone levels, clotting disorders, and a history of infertility.^16^ Moreover, the report indicated that the rate of spontaneous abortion among patients receiving dupilumab (26%) was comparable, or even slightly lower than the general population (30%). In addition, the 4 case reports outlining pregnancy-related complications did not directly attribute the use of dupilumab to these outcomes.

In our systematic review, there were no cases of fetal malformations or congenital defects. Reed et al^33^ described a rare case of congenital defects (panhypopituitarism, hypogonadotropic hypogonadism, a cleft palate, and pharyngomalacia) in the newborn of a patient on dual omalizumab-dupilumab therapy for uncontrolled asthma and not for AD. The patient also experienced uncontrolled severe asthma attacks necessitating oral corticosteroids and intubation. She also developed gestational hypertension and corticosteroid-induced diabetes.^33^ These conditions are strongly associated with an increased risk of congenital defects.^34-37^

The study’s limitations include the small sample sizes in the reviewed studies, which may not be representative of the general population. In addition, some case reports were incomplete in the reporting of outcomes. Insufficient details on maternal, fetal, and labour parameters, along with the inconsistency in the outcome measures of these parameters may have affected the accuracy of our findings. Furthermore, the lack of reporting on ethnicity, comorbidities, concomitant therapies, and other risk factors for pregnancy complications is another limitation.

Overall, this systematic review suggests that dupilumab is not associated with major adverse outcomes in treated pregnant patients or their offspring. Prospective studies with long-term follow-up are essential to assess and confirm the safety of dupilumab in this special population.

## Conclusions

Dupilumab has demonstrated efficacy in treating AD during pregnancy. The absence of major adverse maternal, fetal, and labour-associated outcomes provides reassurance regarding its safety profile. Further studies with extended follow-up periods are needed to solidify these findings, but current evidence supports dupilumab as a potential therapeutic option for pregnant individuals with AD.

## Supplemental Material

sj-docx-1-cms-10.1177_12034754241290806 – Supplemental material for Maternal, Fetal, and Labour Outcomes of Dupilumab Use for Atopic Dermatitis During Pregnancy: A Systematic Review Supplemental material, sj-docx-1-cms-10.1177_12034754241290806 for Maternal, Fetal, and Labour Outcomes of Dupilumab Use for Atopic Dermatitis During Pregnancy: A Systematic Review by Chirag R. Chopra, Mahak Sharma, Mahtab S. Gill, Victoria Del Balso, Noor Sakka and Mohannad Abu-Hilal in Journal of Cutaneous Medicine and Surgery

sj-docx-2-cms-10.1177_12034754241290806 – Supplemental material for Maternal, Fetal, and Labour Outcomes of Dupilumab Use for Atopic Dermatitis During Pregnancy: A Systematic Review Supplemental material, sj-docx-2-cms-10.1177_12034754241290806 for Maternal, Fetal, and Labour Outcomes of Dupilumab Use for Atopic Dermatitis During Pregnancy: A Systematic Review by Chirag R. Chopra, Mahak Sharma, Mahtab S. Gill, Victoria Del Balso, Noor Sakka and Mohannad Abu-Hilal in Journal of Cutaneous Medicine and Surgery
