# Supplementary material for: Maternal, Fetal, and Labour Outcomes of Dupilumab Use for Atopic Dermatitis During Pregnancy: A Systematic Review
Source: J Cutan Med Surg. 2024 Oct 20;29(1):51–5. doi: 10.1177/12034754241290806 (PMC11829506; doi:10.1177/12034754241290806)
Supplement: sj-docx-1-cms-10.1177_12034754241290806 – Supplemental material for Maternal, Fetal, and Labour Outcomes of Dupilumab Use for Atopic Dermatitis During Pregnancy: A Systematic Review [file sj-docx-1-cms-10.1177_12034754241290806.docx]

| **Publication Title** | **Authors** | **Patient Description** | **Pre-Intervention Score (EASI//IGA/DLQI, NRS)** | **Intervention Duration** | **Post-Intervention Score (EASI//IGA/DLQI, NRS)** | **Adverse Effects Reported** |
| --- | --- | --- | --- | --- | --- | --- |
| Atopic Dermatitis Treated Safely with Dupilumab during Pregnancy: A Case Report and Review of the Literature^17^ | Lobo Y.; Lee R.C.; Spelman L. | 36-year-old female (gravida 1 para 1) with severe AD since childhood. | EASI - 67.4 IGA - 4 Diffuse AD affecting 86% of body | Continued to receive dupilumab until 24 weeks and 4 days gestation. Intervention resumed post-partum. | EASI SCORE:  Week 0 of Treatment: 67.4 (severe disease)  Week 16 of Treatment: 1.7 (remission)  EASI score of 40.1 after few weeks of cessation. EASI score of 4.1 6 weeks after resumption of intervention. | Maternal: Gestational Diabetes |
| Severe atopic dermatitis treated successfully with dupilumab throughout pregnancy^38^ | Costley M.; Murphy B. | Female with lifelong history of severe AD. | “Severe” | Uninterrupted | Study reports “symptoms controlled” | No AEs reported |
| Patient with atopic dermatitis, hyper IgE syndrome and ulcerative colitis, treated successfully with dupilumab during pregnancy^19^ | Gracia-Darder I.; Pons De Ves J.; Reyero Cortina M.; Martin-Santiago A. | 28-year-old female with AD diagnosis since age 1. | SCORAD 62 DLQI score of  28/30, NRS-itch 9/10 and NRS-sleep 9/10 | Uninterrupted | At 16th week of treatment:  SCORAD of 21/103  NRS-itch of 3/10.  Study reports skin lesions disappeared, as well as, the respiratory symptoms, while the digestive symptoms did not become worse. | Maternal: Ocular Discomfort |
| The use of dupilumab in severe atopic dermatitis during pregnancy^20^ | Akhtar N.; Khosravi-Hafsehjani T.; Akhtar D.; Dhadwal G.; Kanani A. | 33-year-old woman (1 gravida, 1 para) with severe AD since childhood | IGA 4 (severe Disease) | 2 week break between 27 and 29 weeks of pregnancy | Authors report symptoms “well controlled” with a “severe flare-up” during 2 week break in treatment | Labour-Associated: Caesarean |
| Dupilumab for Atopic Dermatitis During Pregnancy and Breastfeeding: A Case Report^39^ | Alvarenga J.M.; Maria Le A.; Torres T. | 37-year-old woman diagnosed with AD in the first few months of life. Mild until age 30, then worsening | AD lesions affecting 27% area EASI 19.1 SCORAD 54.4 IGA 4 NRS pruritus of 7 | Uninterrupted | After 12 weeks of treatment: EASI 90  NRS pruritus 0  IGA 1 | Maternal: Mild Arthralgias. |
| Case of atopic eczema treated with dupilumab throughout conception, pregnancy, and lactation^40^ | Kage P.; Simon J.-C.; Treudler R. | 36-year-old woman, suffers from atopic eczema since early childhood | SCORAD 51.7 | Uninterrupted | SCORAD between 15 and 18 throughout pregnancy. | No AEs reported |
| A case of atopic eczema treated safely with dupilumab during pregnancy and lactation^41^ | Treudler R.; Kage P.; Simon J.C. | 35-year-old woman, suffers from atopic eczema since early childhood | SCORAD 51.7 | Until 2nd week of pregnancy and resumption at 20th week of pregnancy | SCORAD rose to 30+ within 3 months of treatment pause. Reduced to 11 to 15 after treatment resumption. | No AEs reported |
| Dupilumab for the treatment of severe atopic dermatitis in a pregnant patient: A case report^21^ | Mian M.; Dunlap R.; Simpson E. | A 28-year-old woman with a lifelong history of Atopic Dermatitis (AD) (gravida 3, para 2) | IGA - very severe | From 24th week of pregnancy to post-partum | Authors indicate patient’s skin “dramatically improved” during treatment | Maternal: Eye Irritation, Gestational diabetes  Fetal: Small for gestational age baby |
| Dupilumab for atopic dermatitis during pregnancy and breastfeeding: Clinical experience in 13 patients^42^ | Escola H.; Figueras-Nart I.; Bonfill-Orti M.; Coll-Puigserver N.; Martin-Santiago A.; et al. | 11 pregnant women, 10 Caucasian and 1 Hispanic, between ages of 28 and 41, all suffering from AD for most of their lives. | EASI 30-61 | Uninterrupted (n=5) Partial Pregnancy, between 3 and 8 months (n=6) | Week 16 of treatment:  EASI-75 (83.3% of patients)  Pruritus NRS and DLQI scores were “considerably reduced”  Weeks 24 of treatment:  EASI 75 (90.9% of patients)  Week 52 of treatment:  EASI 75 (90% of patients) | Maternal: Conjunctivitis  Labour-Associated: Dystotic delivery, Caesarean deliveries, Vacuum-assisted delivery  Fetal: Pre-Term birth, Low Birth Weight |
| Assessment Report - Dupixent (EMA)^16^ | N/A | 23 pregnant women (14 patients included in review) | Not Reported | Not Reported | Not Reported | Maternal: Spontaneous abortions |
| Atopic dermatitis treated safely with dupilumab during pregnancy and lactation: A case series of four patients^43^ | Narang Hong, So Yun Park, Hyung Don Kook, Dong Heon Lee, Hye Jung Jung, Mi Youn Park, Jiyoung Ahn | 4 patients age: 33, 35, 39, 29. | Patient 1 EASI: 32  Patient 2 EASI: 16.6 Patient 3 EASI: 35.6 Patient 4 EASI: 14.9 | Intermittent (n=4) | Patient 1 EASI: 1  Patient 2 EASI: 1.6  Patient 3 EASI: 3.4  Patient 4 EASI: 4 | No AEs reported |
| Association between maternal dupilumab exposure and pregnancy outcomes in patients with moderate-to-severe atopic dermatitis: A nationwide retrospective cohort study^44^ | Avallone et al. | 28 women in Italian hospitals, mean age 33.6 (19-45) | Pre-treatment scores (EASI, SCORAD, DLQI) presented in boxplots. Raw data not available. | Intermittent (n=28) | Reduction in EASI, SCORAD, DLQI observed at 3 months, 6 months, and 9 months. | Maternal: Gestational Diabetes, Post-partum Hemorrhage, Oligohydramnios, Miscarriage  Fetal: Pre-Term birth, Respiratory Distress, Pulmonary  Hypertension, Solitary cutaneous mastocytoma, AD symptoms |
| A real-world study of dupilumab in patients with atopic dermatitis including patients with malignancy and other medical comorbidities^45^ | Dea Metko, Maha Alkofide, Mohannad Abu-Hilal | 3 women | PGA 3 or 4 | Uninterrupted (n=3) | PGA 0 or 1 | No AEs reported |

**Supplementary Table 1:** **Summary of Source Publications Reporting AD Symptom Progression and Adverse Effects Associated with Dupilumab Use During Pregnancy.** Pruritus NRS - Pruritus Numeric Rating Scale, EASI - Eczema Area and Severity Index, SCORAD - ("SCORing Atopic Dermatitis"), DLQI - The Dermatology Life Quality Index.

| **AD Onset** | **Number** | **Percent of Sample** |
| --- | --- | --- |
| Prior to Pregnancy | 55 | 80.9% |
| During Pregnancy | 0 | 0% |
| Not Reported | 13 | 19.1% |

**Supplementary Table 2: AD onset timeline in sample population.** Total sample size of 68 pregnant patients.

| **Start of Dupilumab Treatment** | **Number** | **Percent of Reported (n=53)** | **Percent of Sample (n=68)** |
| --- | --- | --- | --- |
| Before Pregnancy | 49 | 92.5% | 72.1% |
| During Pregnancy | 4 | 7.5% | 5.9% |
| Not Reported | 15 | N/A | 22% |
| *Of those that reported start of treatment for the first time during pregnancy (n=4):* | | | |
| **Trimester** | **Number** | **Percent of Reported (n=4)** | |
| 1 | 1 | 25% | |
| 2 | 2 | 50% | |
| 3 | 1 | 25% | |

**Supplementary Table 3: Start of Dupilumab Treatment.** Total sample is 68 pregnant patients, of which 53 patients reported the start of dupilumab treatment. 4 patients began dupilumab treatment for the first time during their pregnancies.

**References**

Costley M, Murphy B. Severe atopic dermatitis treated successfully with dupilumab throughout pregnancy. Clin Exp Dermatol. 2022;47(5):960-961. doi:10.1111/ced.15049.

Alvarenga JM, Maria Lé A, Torres T. Dupilumab for atopic dermatitis during pregnancy and breastfeeding: a case report. Actas Dermosifiliogr. Published online October 17, 2023. doi:10.1016/j.ad.2023.10.005.

Kage P, Simon JC, Treudler R. Case of atopic eczema treated with dupilumab throughout conception, pregnancy, and lactation. J Dermatol. 2021;48(10):E484-E485. doi:10.1111/1346-8138.16033**.**

Kage P, Simon JC, Treudler R. A case of atopic eczema treated safely with dupilumab during pregnancy and lactation. J Eur Acad Dermatol Venereol. 2020;34(6):e256-e257. doi:10.1111/jdv.16235**.**

Escolà H, Figueras-Nart I, Bonfill-Orti M, et al. Dupilumab for atopic dermatitis during pregnancy and breastfeeding: clinical experience in 13 patients. J Eur Acad Dermatol Venereol. 2023;37(9):e1156-e1160. doi:10.1111/jdv.19165**.**

Hong N, Park SY, Kook HD, et al. Atopic dermatitis treated safely with dupilumab during pregnancy and lactation: a case series of four patients. Australas J Dermatol. 2024;65:e100-e103. doi:10.1111/ajd.14255**.**

Avallone G, Cavallo F, Tancredi A, et al. Association between maternal dupilumab exposure and pregnancy outcomes in patients with moderate-to-severe atopic dermatitis: a nationwide retrospective cohort study. J Eur Acad Dermatol Venereol. 2024;38:1799-1808. doi:10.1111/jdv.19794**.**

Metko D, Alkofide M, Abu-Hilal M. A real-world study of dupilumab in patients with atopic dermatitis including patients with malignancy and other medical comorbidities. JAAD Int. 2024;15:5-11. doi:10.1016/j.jdin.2024.01.002**.**
