# Supplementary material for: Maternal, Fetal, and Labour Outcomes of Dupilumab Use for Atopic Dermatitis During Pregnancy: A Systematic Review
Source: J Cutan Med Surg. 2024 Oct 20;29(1):51–5. doi: 10.1177/12034754241290806 (PMC11829506; doi:10.1177/12034754241290806)
Supplement: sj-docx-2-cms-10.1177_12034754241290806 – Supplemental material for Maternal, Fetal, and Labour Outcomes of Dupilumab Use for Atopic Dermatitis During Pregnancy: A Systematic Review [file sj-docx-2-cms-10.1177_12034754241290806.docx]

1

**Supplementary Figure 1.** Flow diagram of literature screening using the COVIDENCE platform and Preferred Reporting Items for Systematic reviews and Meta-Analyses (PRISMA) guidelines. Figure adapted from [http://prisma-statement.org](http://prisma-statement.org/)

**Studies remaining after full-text review**

(n = 13)

**Studies excluded after full-text review**

(n = 5)

Reasons for exclusion:

Duplicates (n=3)

Irrelevant application of intervention (n=1)

No study results (n=1)

**Studies remaining after title/abstract screen**

(n = 18)

**Studies excluded after title/abstract screen**

(n = 101)

**Studies remaining after duplicates removed**

(n = 119)

**Studies excluded due to duplicates**

(n = 166)

**Studies identified in initial search of Ovid EMBASE, MEDLINE, and PubMed databases**

(n =285)

Identification

Screening

Eligibility

Included

Primary original research studies such as case reports, case series, randomized controlled trials, prospective and retrospective studies, and cross-sectional studies were included. Exclusion criteria comprised review articles, book chapters, opinion pieces, conference/poster abstracts, and theses. Unpublished studies were not sought. Inclusion criteria included pregnant patients who were treated with dupilumab. Exclusion criteria included non-pregnant patients who were treated with dupilumab, pregnant patients who were treated with dupilumab only during the preconception or postpartum period, and pregnant patients treated with dupilumab for non-dermatological concerns.
